# Characterization of HIV risks in a Brazilian sickle cell disease population

**DOI:** 10.1186/s12889-020-09702-5

**Published:** 2020-10-23

**Authors:** P. F. Blatyta, S. Kelly, T. T. Goncalez, A. B. Carneiro-Proietti, T. Salomon, C. Miranda, E. Sabino, L. Preiss, C. Maximo, P. Loureiro, B. Custer, C. de Almeida-Neto, for the NHLBI Recipient Epidemiology and Donor Evaluation Study-III (REDS-III) International Component, Brazil

**Affiliations:** 1 Hospital Moysés Deutsch, São Paulo, SP Brazil; 2 Disciplina de Ciências Médicas, Faculdade de Medicina da Universidade de São Paulo, São Paulo, SP Brazil; 3 Vitalant Research Institute, San Francisco, CA USA; 4 Fundação Hemominas, Belo Horizonte, MG Brazil; 5 Instituto de Medicina Tropical da FMUSP, São Paulo, SP Brazil; 6 Research Triangle Institute, International, Rockville, MD USA; 7 Hemorio, Rio de Janeiro, RJ Brazil; 8 Fundação Hemope and Universidade de Pernambuco, Recife, PE Brazil; 9 Fundação Pró-Sangue de São Paulo, São Paulo, SP Brazil

**Keywords:** HIV, Sickle cell disease, Transfusion transmitted infections, HIV risk factors

## Abstract

**Background:**

A low prevalence of HIV in sickle cell disease (SCD) patients has been reported in the literature though mechanisms for this are not understood.

**Methods:**

HIV risk behaviors were compared between SCD cases and non-SCD controls using a self-administered audio computer-assisted self-interview. SCD cases were recruited from a multi-center SCD cohort established in Brazil; controls were recruited from SCD social contacts. Categorical variables were analyzed using Chi-Square or Fisher exact test. Continuous variables were compared using the Mann-Whitney U test.

**Results:**

There were 152 SCD cases and 154 age/location matched controls enrolled at three participating Brazilian centers during 2016–17. No significant differences in number of sexual partners (lifetime or previous 12 months), male-to-male sex partners or intravenous drug use were observed. Cases received more transfusions, surgeries, and acupuncture treatment.

**Conclusions:**

Besides the risk of transfusion-transmitted HIV, which is now exceedingly rare, SCD and non-SCD participants demonstrated similar HIV risk behaviors. Causes other than risk behaviors such as factors inherent to SCD pathophysiology may explain the reported low prevalence of HIV in SCD.

**Supplementary information:**

The online version contains supplementary material available at 10.1186/s12889-020-09702-5.

## Background

The limited literature focused on human immunodeficiency virus (HIV) in sickle cell disease (SCD) demonstrates that HIV is relatively rare in this population. Studies over the past few decades have consistently shown a lower prevalence of HIV in SCD compared to non-SCD populations [1–4], however these studies were limited by small numbers of study participants, insufficient matching of control populations and no measurement of HIV risk factors.

Possible explanations for the reduced rates of HIV in SCD may include inhibition of human immunodeficiency virus (HIV) replication due to the pro-inflammatory status of SCD patients [5], influence of Duffy antigen on HIV-1 capability of infecting SCD host cells [6], HIV-1 transcription and replication inhibition in SCD patients’ cells due to increased ferroportin expression and reduced intracellular iron [7], or an increased expression in SCD of CCR5Δ32 [8] that promotes resistance to HIV infection. Risk of HIV in SCD may be also modulated by lower exposure to HIV, as a few prior studies have demonstrated lower levels of sexual activity in small samples of SCD patients [9–11]. Individuals with SCD may suffer from chronic pain, priapism [12] and other conditions that could potentially affect sexual health.

To evaluate whether the apparent lower HIV prevalence in SCD could be related to decreased HIV risk behaviors in this population, this study compared HIV risk behaviors between adult SCD participants (cases) and age/location matched non-SCD controls. The primary outcome was the number of sexual partners in the previous year, compared between cases and controls; other HIV risk factors included male-to-male sex, number of lifetime sexual partners, use of condoms and other sexual practices, age of sexual debut, intravenous drug use (IDU), history of blood transfusions, and sex with an individual known to be HIV-positive or at high risk for HIV.

## Methods

### Study design and procedures

This is a nested case-control study of the Brazilian part of a multi-center Recipient Epidemiology and Donor Evaluation-III (REDS-III) program supported by the National Heart Lung and Blood Institute (NHLBI) of the Unites States of America (USA) National Institutes of Health. The REDS-III program researches on blood safety, blood availability and impact of transfusion in patients in the USA, Brazil, China, and South Africa. The REDS-III Brazil SCD cohort study was designed to investigate SCD pathogenesis and the impact of transfusion on disease outcomes and was a cooperation between Vitalant Research Institute in San Francisco, CA and six participating centers in Brazil. Research Triangle Institute, International, served as the data coordinating center.

Details of the cohort study procedures and enrollment findings have been described previously [13]. Briefly, patients with at least one clinical encounter in the last 3 years were randomly selected as eligible. Participating centers were in the southeast cities of Belo Horizonte, Rio de Janeiro, Sao Paulo, Juiz de Fora, Montes Claros, and the northeast city of Recife. Patients were recruited and enrolled at routine patient care visits from 2013 to 2015 to establish the cohort and follow up visits were conducted through 2018.

SCD cases for this analysis of HIV risk factors were selected from the adult cohort participants (aged 18 years or older) at the three largest centers in Rio de Janeiro, Belo Horizonte, and Recife. A list of all adult cohort participants at these three sites was randomly ordered and potential subjects were recruited in the order of this list. The list was divided in blocks of 10 participants to be contacted simultaneously and up to 6 attempts were made to contact each participant in a block before moving to the next block in the randomly ordered list. There were no exclusion criteria for cases. After recruitment of cases, the controls were recruited matched on location and were age-matched to cases using a ± 5-year window around each case.

SCD advocacy groups were contacted at participating sites to identify appropriate communities to target for recruitment of controls. These included friends and family of SCD patients in waiting rooms as well as employees of participating centers. Exclusion criteria for controls included diagnosis of SCD by self-report, which was confirmed by performing high performance liquid chromatography (HPLC).

A questionnaire with sexual and other risk behaviors related to HIV infection (Additional file 1) was administered to participants using a self-administered audio computer-assisted self-interview (ACASI- QDS Software, Nova Research Co, Bethesda, MD). A trained assistant was available to facilitate the use of ACASI instrument features, such as answering basic questions and troubleshooting technical issues related to the study questionnaire. However, participants could read or listen to the questions and respond privately. The ACASI has previously been used and validated [14] in the Brazilian population for interviewing participants regarding potentially stigmatized behaviors such as drug use and sexual preferences through prior REDS research to identify HIV risk factors in blood donors [15–17].

All cases and controls were tested for HIV using the Architect Ag/Ab by Abbott as a screening test and confirmed with the Genscreen ultra HIV Ag/Ab Elisa by Biorad. Any identified HIV positive participants were referred for appropriate counseling and treatment at the participating center. Controls were tested for SCD using HPLC to detect hemoglobin variants. Controls that were diagnosed with HbS or HbC trait were counseled about the carrier state but not excluded as a control from the study. If consent was provided, any remaining blood after testing was complete was transferred to a Biobank at Faculdade de Medicina da Universidade de Sao Paulo for future use in research.

Approval was obtained from the USA Institutional Review Board (IRB) of record at the University of California San Francisco, data coordinating center IRB and ethical committees at the three participating Brazilian hemocenters and the Brazilian National Ethical Committee (CONEP), approval number 46981615.0.1001.0065. All participants signed informed consent.

### Statistical considerations

The primary outcome was the number of sexual partners in the previous year, compared between cases (SCD) and controls (non-SCD). Other HIV risk factors ascertained by ACASI included male-to-male sex, number of lifetime sexual partners, use of condoms and other sexual practices, age of sexual debut, intravenous drug use (IDU), history of blood transfusions, and sex with an individual known to be HIV-positive or at high risk for HIV.

Summary statistics are presented as frequencies and percentages for categorical variables, and interquartile ranges (IQR) for continuous variables. Categorical variables were analyzed using Chi-Square, or Fisher exact test when appropriate. Continuous variables were compared using the Mann-Whitney U test. Two-sided *p*-values < 0.05 were statistically significant. All statistical analyses were performed using SAS 9.4.

### Sample size justification

A simulation study was conducted to explore statistical power for a comparison of the primary outcome, rates of sexual activity in the prior year among patients with sickle cell disease (cases) and age matched persons without sickle cell disease (controls). It was assumed that subjects would be classified into three levels of sexual activity: 0 (zero activity group), 1–2 (low activity group) or 3 and more partners (high activity group) in the preceding year to produce a 3-level multinomial distribution. The presumed distribution in the controls was based on a previous REDS-II study of HIV negative blood donors in Brazil [18] that identified a distribution of 6.3% in the zero activity group, 86.6% in the low activity group and 7.1% in the high activity group. Considering the previously cited study surveying SCD females and non-SCD controls that demonstrated 39% of SCD participants were sexually active compared to 81% of controls [11], an assumption of differences on the order of magnitude of 11, 88 and 1% (0, 1–2, 3+ partners, respectively) in the cases compared to the presumed distribution of 6.3, 86.6, 7.1% in controls is reasonable. As 80% power is achieved with approximately 150 subjects per group in this distribution, this number was chosen as the sample size.

## Results

### Demographics of study population

There were 2793 participants (1234 adults) included in the REDS-III Brazil SCD cohort. The number of adults identified as eligible in the three centers participating in this project at Hemominas (Belo Horizonte), Hemorio (Rio de Janeiro), and Hemope (Recife) was 427, 352, and 313, respectively. There were 7 contacted patients who declined participation and 63 (Hemominas), 40 (Hemorio), and 50 (Hemope) SCD patients consented to participation. There was one patient who did not complete the entire questionnaire; therefore 152 cases were included in this analysis. There were 154 non-SCD controls enrolled. The number of controls did not match exactly the number of cases because two hemocenters recruited 2 controls at the exact time the recruiting was finished.

The average age of all participants was 34.5 years (range 18–69 years) and 165 (53.9%) females, and 141 (46.1%) males participated. Most participants reported being single, having a highest educational level of elementary/middle school and a monthly income of ranging from 701 to 1400 Brazilian reais (approximately US$219–437 dollars in 2016). There were no significant differences in the gender or age distribution, nor any of the socioeconomic variables between the cases and controls (Table 1). There were significant differences in self-reported race and current employment status between the two populations. While mixed race was most commonly reported by both SCD patients and non-SCD controls (slightly over half of both groups), more SCD cases self-reported black race (34.8%) when compared to controls (16.9%) and more controls self-reported white race (24% compared to 10.5%, *p* = .0003). Unemployment rate was higher among SCD patients when compared to controls (28.3% vs. 7.8%, *p* = .0002).

**Table 1:** Demographic data of SCD cases and non-SCD controls

	SCD patients (cases = 152)		Non- SCD participants (controls = 154)		*p* value
Blood Center Affiliation	**n**	**column%**	**n**	**column%**	
Hemope	50	32.9	50	32.4	.7
Hemorio	39	25.7	46	29.9	
Hemominas	63	41.4	58	37.7	
Gender					
Male	73	48.0	68	44.2	.5
Female	79	52.0	86	55.8	
Education level					
Never attended	2	1.3	0	0.0	.3
Elementary/ Middle School	124	81.6	122	79.2	
Adult Literacy/Technical school	8	5.3	14	9.1	
Bachelors/Masters/Doctorate	18	11.8	18	11.7	
Ethnicity					
White	16	10.5	37	24.0	<.001
Black	53	34.8	26	16.9	
Indigenous (Indian)	1	0.7	1	0.6	
Mixed	79	52.0	84	54.6	
Unknown	1	0.7	0	0.0	
Other	2	1.3	6	3.9	
Age					
18 to < 25 years	32	21.0	27	17.6	.8
25 to < 35 years	49	32.2	44	28.8	
35 to < 45 years	45	29.6	51	33.3	
45 to < 55 years	19	12.5	24	15.7	
> 55 years	7	4.6	7	4.6	
Marital status					
Single	75	49.3	56	36.4	.1
Married	40	26.3	58	37.7	
Divorced/ Widowed	12	7.9	13	8.4	
Living together	25	16.5	27	17.5	
Occupation					
Never worked / No occupation	43	28.3	12	7.8	<.001
Currently working	109	71.7	142	92.2	
Monthly income					
< R$1400	106	69.7	97	63	.6
R$1401- R$6000	43	28.3	51	33	
> R$6000	3	2.0	5	3.3	
Don’t know	0	0.0	1	0.7	

All SCD cases demonstrated non-reactive serologic testing for HIV. One control was confirmed to be HIV positive with reactive screening and confirmatory assays. HPLC testing confirmed that no controls had SCD, although 37 carriers with Hemoglobin S (HbS) trait and 2 with Hemoglobin C (HbC) trait were identified.

There was no difference between SCD and non-SCD participants in the primary outcome, with both groups reporting a median of one sexual partner in the previous 12 months (*p* = .8). Most participants (70.4% of SCD cases and 77.3% of non-SCD controls) disclosed 1–2 sexual partners, with fewer participants reporting no partners (15.1% of cases and 10.4% of controls) or three or more partners (12.5% of cases and 11.0% of controls) (Table 2). Most participants reported being straight/heterosexual (87.5% of cases and 89.6% of controls) and had a median of three lifetime sexual partners. Having partners of same gender did not differ among the two groups. The age of sexual initiation was older among female cases compared to their controls (20 years versus 17.5 years; p.001). The number of episodes of vaginal and anal intercourses and use of condoms during sex did not differ between female cases and controls. Receptive and insertive anal sex and the use of condoms among males were also similar between the two groups (Table 2).

**Table 2:** Sexual risk factors for HIV infection among SCD cohort

	SCD patients (cases = 152)		Non- SCD participants (controls = 154)		*p* value
Primary outcome	**n/median**	**%**	**n /median**	**%**	
Number of sexual partners in previous year (12 months)					
- 0	23	15.1	16	10.4	3
- 1–2	107	70.4	119	77.3	
- 3 or more	19	12.5	17	11.0	
Don’t Know/ Refuse to Answer	3	2.0	2	1.3	
	**n/median**	**IQR**	**n/median**	**IQR**	
Number of sexual partners in the previous year (12 months)	1	(1)	1	(1)	.8
Secondary outcomes					
Number of lifetime partners	3	(1–9)	3	(1–8)	.8
Number of lifetime female partners (for men only)	5.5	(3–14)	5	(3–15)	.9
Age of sexual debut with a woman (for men only)	18	(16–19)	17	(15–19)	.1
Number of lifetime male partners (for men only)	0	(0–0)	0	(0–0)	.3
Age of sexual debut with a man (for men only)	20	(16–29)	15	(13–18)	.1
Number of lifetime male partners (for women only)	2	(1–5)	2	(1–3)	.1
Age of sexual debut with a man (for women only)	20	(17–22)	17.5	(16–19)	.001
Use of condoms with vaginal sex (female only)	**n/median**	**%**	**n/median**	**%**	
-Never	17	29.8	18	25.7	.8
-Sometimes	26	45.6	36	51.4	
-Every time	14	24.6	16	22.9	
Number of anal sex intercourses in past 12 months (female only)					
-None	64	81.0	58	67.4	.08
-1 to 3 times	12	15.2	17	19.8	
-4 to 10 times	3	3.8	4	4.7	
-More than 10 times	0	0.0	5	5.8	
-Don’t Know/ Refuse to Answer	0	0.0	2	2.3	
Use of condoms with anal sex (female only)					
-Never	7	46.7	17	60.7	.7
-Sometimes	6	40.0	8	28.6	
-Every time	2	13.3	3	10.7	
Number of insertive anal sex intercourses in past 12 months (males)					
-None	47	64.4	41	60.3	.8
-1 to 3 times	11	15.1	15	22.1	
-4 to 10 times	9	12.3	6	8.8	
-More than 10 times	5	6.8	5	7.3	
-Don’t Know/ Refuse to Answer	1	1.4	1	1.5	
Use of condoms with insertive anal sex					
-Never	9	34.6	7	25.9	.9
-Sometimes	12	46.1	13	48.2	
-Every time	5	19.2	6	22.2	
-Don’t Know/ Refuse to Answer	0	0.0	1	3.7	
Number of receptive anal sex intercourses in past 12 months (male)					
-None	70	95.9	63	92.7	.3
-1 to 3 times	2	2.7	2	2.9	
-4 to 10 times	0	0.0	0	0.0	
-More than 10 times	1	1.4	0	0.0	
-Don’t Know/ Refuse to Answer	0	0.0	3	4.4	
Use of condoms with receptive anal sex (male)					
-Never	1	33.3	1	20.0	.7
-Sometimes	1	33.3	0	0.0	
-Every time	1	33.3	2	40.0	
-Don’t Know/ Refuse to Answer	0	0.0	2	40.0	
Sexual orientation					
-Straight/heterosexual	133	87.5	138	89.6	.6
-Bisexual	5	3.3	4	2.6	
-Gay/lesbian/homosexual	2	1.3	5	3.2	
-Something Else	7	4.6	4	2.6	
-Don’t Know/ Refuse to Answer	5	3.3	3	2.0	
Male to male partners					
-Yes	1	1.4	2	2.9	.6
-No	72	98.6	66	97.1	
Female to female partners					
-Yes	3	3.8	2	2.3	.7
-No	76	96.2	84	97.7	

Sex with a person known to be HIV positive or exhibiting HIV risk behaviors [IDU, man who has had sex with another man (MSM), history of blood transfusion] did not differ among cases and controls (data not shown).

SCD cases were more likely to receive blood transfusions (83.5% vs. 1%, *p* = .0001) and reported higher prevalence of surgical procedures (69.7% of cases vs. 52% of controls, *p* = .001) and acupuncture treatments (9.9% versus 1.3%, *p* = .0004) when compared to controls (Table 3).

**Table 3:** Other risk factors for HIV infection among SCD cohort

	SCD (cases) *N* = 152		Non- SCD (controls) *N* = 154		*p* value
	Median	IQR	Median	IQR	
Number of previous transfusions	8	2–15	1	1–1	<.001
	**Yes**	**%**	**Yes**	**%**	
Previous blood transfusion	127	83.5	8	5.2	<.001
Previous transplant	1	0.7	0	0.0	.5
Previous surgery	106	69.7	80	52.0	.001
Previous tooth extraction	126	82.9	119	77.3	.2
Born to an HIV positive mother	0	0.0	1	0.6	.7
Breastfed by an HIV positive person	1	0.7	0	0.0	.1
Three or more days spent in prison	2	1.3	2	1.3	.99
Previous acupuncture treatment					
-Yes	15	9.9	2	1.3	<.001
-Don’t know/ refuse to answer	2	1.3	0	0.0	
Number of tattoos					
-0	130	85.5	129	83.8	.3
-1 to 2	13	8.5	20	13.0	
-3 or more	8	5.3	5	3.2	
Where the tattoo was done (most recent)					
-Tattoo parlor	18	81.8	14	56.0	.06
-At home, a friend’s place, or at parties/raves	4	18.2	11	44.0	
Ear or body piercings					
-1 or 2	19	12.5	10	6.5	.1
-3 or more	4	2.6	2	1.3	
Work with care of humans or body fluids	10	22.7	38	36.9	.2
Needle stick injury or sharp object	9	20.4	20	19.4	.8
Human secretions splashed into eyes, mouth or nose	1	2.3	9	8.7	.3

A higher proportion of SCD cases reported not drinking alcohol (59.9%) compared to controls (42.2%, *p* = .006). No cases and only one control disclosed use of intravenous drugs and there were no significant differences between use of other drugs or sharing of needles between the two populations (Table 4).

**Table 4:** Alcohol, non-injected and injected illegal drug use among the cohort

	SCD (cases = 152)		Non- SCD (controls =154)		*p* value
	n	%	n	%	
Alcohol ingestion					
-Never	91	59.9	65	42.2	.006
-1–3 times per month or less	45	29.6	52	33.8	
-1–2 times per week	11	7.2	23	14.9	
-3–6 times per week	5	3.3	11	7.1	
-Everyday	0	0.0	3	2.0	
	**n/ median**	**IQR**	**n/ median**	**IQR**	
Number of drinks	3	2–5	3	2–6.5	.9
Use of any non-injected illegal drugs	**25**	16.4	19	12.3	.3
Share of pipes or straw with another person					
-Never	10	40.0	9	47.4	.7
-Sometimes	11	44.0	9	47.4	
-Always	3	12.0	1	5.2	
-Don’t know/ refuse to answer	1	4.0	0	0.0	
Use of illegal injection drugs	0	0.0	1	0.6	.9
Type of illegal drugs used*					
-Cocaine	0	0.0	1	100.0	ƚ
-Mushrooms	1	4.0	1	5.3	.8
-LSD	0	0.0	3	15.8	.07
-Other	4	16.0	1	5.3	.4
Shared needles or syringes to inject non-prescription substances	1	4.2	1	4.3	.99

*The columns don’t add up to 100% because many participants declared more than one type of drug use

ƚOnly one participant declared use of cocaine; not enough observations to compute a *p*-value

## Discussion

This study sought to determine if there was a difference in HIV risk behaviors between persons with SCD and a similar non-SCD control population that could explain the lower prevalence of HIV in SCD. We found no difference between SCD patients and controls in the number of sexual partners (lifetime or partners in last year) and no difference in other key HIV risk factors such as MSM, IDU or sex with person known to be HIV positive or at high risk for HIV.

To our knowledge, this is the largest study to quantify HIV risk in the SCD population. Two other smaller studies analyzing risk factors related to sexual activity in SCD have been reported. One survey administered to 52 female SCD patients and 80 female non-SCD controls in 1984 demonstrated that female SCD patients were less likely to be sexually active and more likely to have an older age of sexual debut [11]. A separate study of 120 SCD adolescents in Jamaica compared to the general Jamaican adolescent population found no difference in sexual activity between SCD and the general population, but did find older age of sexual initiation in females with SCD compared to the control females [10]. These studies did not differentiate sex with male vs. female partners. Consistent with these studies, we found the age of sexual initiation with a male partner to be older in female SCD patients compared to female non-SCD controls.

The SCD patients in the present study disclosed similar prevalence of sharing of pipes/needles as the control population but demonstrated reduced ingestion of alcohol. This finding contrasts with the study from Jamaica, which identified that a larger proportion of SCD adolescents had ingested alcohol compared to non-SCD controls [10]. None of our SCD patients reported intravenous drug use, consistent with the Jamaican study.

As expected, our SCD patients were more likely to have received red blood cell transfusions than controls. However, the risk of HIV acquisition due to transfusion is exceedingly rare with current strategies to screen blood donors for HIV. We have shown the residual risk of HIV transfusion transmission to be as low as approximately 4.2 in 1 million transfusions with current testing strategies in Brazil [19]. We also believe the increased exposure of SCD patients to surgeries and acupuncture treatments did not increase the risk of acquiring HIV [20], as those procedures are practiced under sanitary procedures with sterilized equipment [21] at the treating medical facilities. The higher exposure to acupuncture among cases was presumed to be a pain control method in our SCD population.

Although our case and control population had a relatively similar demographic profile, one significant difference was that almost a third of SCD patients were not working. This is consistent with previous studies in the United States, Brazil and other countries that have reported unemployment rates of 25–81% among SCD adults [22, 23]. The frequent medical visits and hospitalizations, as well as pain episodes contribute to missing work days and potentially neurocognitive effects of SCD all likely to contribute to this finding [24].

Our study has limitations. While the cases and controls were similar in many aspects, there were differences in rates of unemployment (higher in SCD) and self-reported race (more likely to be black in SCD) that may have introduced bias in the analysis. Previous reports have shown HIV prevalence is higher for individuals in lower socioeconomic status and non-white populations [25]. However, the monthly income was not different between the two groups, presumably because of social programs to provide income and assistance, and the imbalance in race does not alter our study’s conclusion of similar HIV risk behaviors between SCD and non-SCD participants. An additional limitation is that we cannot ascertain the data obtained from the participants on drug abuse and alcohol are completely honest, as those practices were self-reported, and it is possible that the subjects did not feel comfortable admitting to those behaviors.

We elected to study the risk of HIV in SCD as studies over the past few decades have demonstrated a lower prevalence of HIV in SCD compared to a non-SCD population [1–4], and it is unknown if the mechanism for the apparent lower HIV prevalence is related to decreased HIV risk behaviors in SCD populations or some ability of SCD pathophysiology to limit HIV transmission and/or progression. Data to support the latter has included a study of national discharge survey data of adult African-Americans in the United States which demonstrated a lower risk of HIV and SCD co-morbidities, but not SCD and HCV, which has similar risk factors for acquisition [26]. In addition, other reports have suggested improved HIV outcomes in SCD. A previous study from USA compared 18 SCD patients with HIV to 36 non-SCD controls with HIV. Cases and controls were matched for age, race and gender [5]. Eight out of 18 (44%) of SCD cases were long-term non-progressors (LTNP), while in the control group 5 out of 36 (13.9%) were LTNP, with 10 years of mean follow up (*p* = .0193). AIDS was the cause of death in 5 out of 18 (23%) SCD patients with HIV, while it caused the death in 22 out of 36 (61%) of controls with HIV only. Our group has recently explored the epidemiology of HIV transmission by retrospectively reviewing the Transfusion Safety Study [27] to compare HIV status between SCD and other congenital anemia patients who were routinely exposed to blood products during the high-risk period before HIV screening implementation [28]. Congenital anemia patients demonstrated a higher risk of HIV acquisition compared to SCD (odds ratio 13.1; 95% confidence interval 1.6–108.9).

Hypothesized or tested biologic mechanisms that might explain the impact of SCD pathophysiology on HIV include an inhibition of HIV replication due to the immunologic changes and pro-inflammatory component of SCD pathophysiology, differences in quantitative expression of HIV receptors [6] or expression of alleles that promotes receptor resistance to HIV infection [8] in SCD populations. Kumari et al. recently demonstrated decreased HIV infectivity in SCD T cells in vitro. Their work showed that increased iron export by ferroportin may restrict HIV infection in SCD via up-regulation of SAMHD [7] (sterile alpha domain and histidine-aspartic domain containing protein-1).

## Conclusions

In summary, our results suggest SCD cases and controls had similar HIV related risk behaviors; our findings support the concept that SCD patients may be protected from HIV infection due to characteristics inherent to SCD pathophysiology, as suggested by the Kumari study. Further studies on the prevalence of HIV and its interaction with SCD are necessary to elucidate the relationship between the two diseases. Identifying the underlying mechanism of potential HIV resistance is not only critical for persons with SCD, but also for identification of methods of HIV resistance in general which could lead to exploration of novel therapeutic targets.

## Supplementary information

Additional file 1. ACASI questionnaire. (DOCX 34.8 kb)

## Data Availability

the datasets used and/or analyzed during the current study are available from the corresponding author on reasonable request.

## Abbreviations

HIV: Human Immunodeficiency Virus
REDS-III: Recipient Epidemiology and Donor Evaluation Study-III
NHLBI: National Heart Lung and Blood Institute
SCD: Sickle cell disease
CCR5Δ32: 32-bp deletion in chemokine (C-C motif) receptor 5 (CCR5)-tropic (R5) strain
IDU: Intravenous drug use
USA: Unites States of America
HPLC: High performance liquid chromatography
ACASI: Self-administered audio computer-assisted self-interview
IRB: Institutional Review Board
IQR: Interquartile ranges
HbS: Hemoglobin S
HbC: Hemoglobin C
MSM: Man who has sex with another man
LTNP: Long-term non-progressors
SAMHD: Sterile alpha domain and histidine-aspartic domain containing protein-1
