# Supplementary material for: Characterization of HIV risks in a Brazilian sickle cell disease population
Source: BMC Public Health. 2020 Oct 23;20:1606. doi: 10.1186/s12889-020-09702-5 (PMC7585195; doi:10.1186/s12889-020-09702-5)
Supplement: Supplementary file 1 — ACASI questionnaire. (DOCX 34.8 kb) [file 12889_2020_9702_MOESM1_ESM.docx]

**Additional file 1: ACASI questionnaire**

REDS-III SCD

**Characterization of risk of HIV and HIV outcomes in the Brazilian Sickle Cell Disease (SCD) population and comparison of SCD outcomes between HIV sero-positive and negative SCD”**

2014 (English)

| Public reporting burden for this collection of information is estimated to average 45 minutes per response, including the time for reviewing instructions, searching existing data sources, gathering and maintaining the data needed, and completing and reviewing the collection of information. **An agency may not conduct or sponsor, and a person is not required to respond to, a collection of information unless it displays a currently valid OMB control number.** Send comments regarding this burden estimate or any other aspect of this collection of information, including suggestions for reducing this burden, to: NIH, Project Clearance Branch, 6705 Rockledge Drive, MSC 7974, Bethesda, MD 20892-7974, ATTN: PRA (0925-NEW). Do not return the completed form to this address. |
| --- |

**OMB Number: 0925-XXXX**

**Expiration Date: XX/XX/XXXX**

SECTION A - STUDY DATA

This section is to be completed by the research assistant or other research staff.

A1. Study ID

__ __ __ __ __ __ __

A2. Hemocenter ID

__ __ __ __ __ __ __ __

A3. Mark if subject is case or control

____ Case

_____ Control

A4. Hemocenter (Choose one)

31 HEMOPE - Pernambuco

32 FPS - Sao Paulo

33 HEMOMINAS - Minas Gerais

35 Hemominas – Juiz de Fora

36 Hemominas – Montes Claros

34 HEMORIO-Rio de Janeiro

A5. Month of interview (Choose one)

01 January

02 February

03 March

04 April

05 May

06 June

07 July

08 August

09 September

10 October

11 November

12 December

A6. Year of interview (please enter four numbers)

__ __ __ __ yyyy

A7. Research Assistant Initials: __ __ __ __ __

A8. Study subject is able to read (Choose one)

0 No

1 Yes

7 Don't Know

8 Refuse to Answer

If study subject is not already sitting at the computer, at this time please make sure the study subject is sitting at the computer and has put the headphones on.

SECTION B - DEMOGRAPHIC DATA

This study has been approved by Ethical Committees in Brazil and the USA. This study also has been approved by the Office of Management and Budget; OMB: XXXXX, OMB approval expires: XXXXX

We are asking these questions to understand if the risk of HIV in people with sickle cell disease is different than people without sickle cell disease. We will ask many personal questions. Please answer the questions as honestly as you can. We will not tell anyone your answers to the questions. When we analyze and report the answers, your answers will be grouped together with other peoples’ answers. This means anyone reading the results of this study will only know the answers of the entire group together, not your individual answers.

In this first section the research assistant will show you how to use the computer to answer the questions. After the research assistant helps you finish this section, you will be left to complete the interview in private. If you have any questions at anytime or are confused, please ask for help.

B1. **What is your gender?**

1 Male

2 Female

3 Transgender

7 Don’t Know

8 Refuse to Answer

B2. What is your birth year?

__ __ __ __

9997 Don’t Know

9998 Refuse to Answer

B3. What is your birth month? (Choose one)

__ __ January

__ __ February

__ __ March

__ __ April

__ __ May

__ __ June

__ __ July

__ __ August

__ __ September

__ __ October

__ __ November

__ __ December

__ __ Don’t Know

__ __ Refuse to Answer

B4. What is your birthday?

__ __

97 Don’t Know

98 Refuse to Answer

B5a. What is your country of birth? (Choose one)

1 Brazil

2 Other

7 Don’t Know

8 Refuse to Answer

***If B5a is not equal to 2, then skip to instruction before B6.***

B5b. Please specify your country of birth

__ __ __ __ __ __ __ __ __ __ __ __ __ __ __ __ __ __ __ __ __ __ __ __ __ __ __ __ __ __ __ __ __ __ __ __ __ __ __ __ __ __ __ __ __ __ __ __ __ __

From now on, you will be left alone to answer these questions in privacy. Please, if you have any questions call the research assistant for help.

B6. What is your race? (Choose one)

1 Caucasian

2 Black

3 Asian

4 Mixed

5 Indian

6 Other

7 Don’t Know

8 Refuse to Answer

***If B6a is not equal to 6, then skip to instruction before B7.***

B6b. Please specify your race

__ __ __ __ __ __ __ __ __ __ __ __ __ __ __ __ __ __ __ __ __ __ __ __ __ __ __ __ __ __ __ __

B7. What is your current marital status? (Choose one)

1 Single, never married.

2 Living together, not legally married.

3 Married.

4 Separated/divorced.

5 Widowed.

7 Don’t Know

8 Refuse to Answer

***If B7 is not equal to 2 or 3, then skip to B9.***

B8. If you are married or living with someone as your partner, is this person:

1 Male

2 Female

7 Don’t Know

8 Refuse to Answer

B9. What is the highest level of education you have completed? (Choose one)

00 Attended but did not complete primary school

01 Primary school

02 Middle school

03 Adult literacy school

04 Technical school

05 College

06 Masters

07 Doctorate

97 Don’t Know

98 Refuse to Answer

B10 What is your occupation ? (now or in the past. If you had more than 1 occupation, choose the occupation you had for the longest time period)

( ) Never worked / No occupation

( ) Housewife / Owner of house (organizing, cleaning, cooking, shopping for the home)

( ) Business / information technology / computer programming / finance / accounting

( ) Research science (biotechnology, biologist, physicist, chemist, psychologist, epidemiologist, sociologist)

( ) Education (teacher, teaching assistant, principal)

( ) Art / entertainment (artist, designer, singer, dancer, writer, television/film, photographer)

( ) Heathcare (Doctor, dentist, nurse, home health aide, dental assistant, nursing aide, phlebotomist, lab technician)

( ) Military or Protective services (police, firefighter, detective, security guard)

( ) Food preparation and related services (cooks, waiters, waitresses, bartenders, dishwasher)

( ) Building or grounds cleaning and maintenance (house cleaning, janitor, landscaping)

( ) Personal care occupations (hairdresser, manicure, pedicure)

( ) Sales (cashiers, real estate, salesperson)

( ) Office and administrative upport (lawyer, secretary, office administration, front desk of business or hotel)

( ) Rural work (agriculture, fisheries)

( ) Construction or Maintenance/Repair (building roads, houses, offices, mechanic, plumber)

( ) Transportation (bus or taxi driver, delivery person)

( )Outros Other

Specify other occupation of the patient ______________________________

**B11** What is the approximate monthly income of your family

( )Less than R$ 700,00

( )Between R$ 701,00 and R$ 1.400,00

( )Between 1.401,00 and R$3.000,00

( )Between R$ 3.001,00 and R$ 6.000,00

( )More than R$ 6.000,00

( )Unknown

( )Refuse to respond

Section C - Previous HIV testing

C1.

Question shown for cases: Have you ever been tested for HIV for any reason other than the routine care of your sickle cell disease?

Questions shown for controls: Have you ever been tested for HIV

0 No ***Skip to C2***

1 Yes

7 Don't Know

8 Refuse to Answer

C1a. What was the reason for the HIV test? (Choose one)

1 Pregnancy care

2 To get health insurance

4 I wanted to know my HIV status

5 Other

7 Don't Know

8 Refuse to Answer

***If C5a is not equal to 5, then skip to C2.***

C1b. Please specify the other reason for test

__ __ __ __ __ __ __ __ __ __ __ __ __ __ __ __ __ __ __ __ __ __ __ __ __ __ __ __ __ __ __

C2. Have you had an HIV test at any other place outside the Hemocenter?

0 No ***Skip to instruction before D1***

1 Yes

7 Don't Know

8 Refuse to Answer

C2a. What other place outside the Hemocenter did you have an HIV test? (Mark all that apply)

1 Private lab

2 Health department

3 Hospital

4 Public lab

5 Other test site

7 Don't Know

8 Refuse to Answer

***If C2a is not equal to 5, then skip to instructions before D1.***

C2b. Please specify the other test site.

__ __ __ __ __ __ __ __ __ __ __ __ __ __ __ __ __ __ __ __ __ __ __ __ __ __ __ __ __ __ __

Section D - Sexual History

This section of the questionnaire will ask you some questions about sex. We understand that these questions are personal and could be hard to answer. Please remember that the questions are part of a scientific study. This form does not have your name (only a code), so even if someone sees the answers on the form, the answers cannot easily be connected to you. Please answer these questions to the best of your knowledge and as honestly as you can.

D1. What do you consider yourself to be? (Choose one)

1 Straight/heterosexual

2 Bisexual

3 Gay/homosexual

7 Don't Know

8 Refuse to Answer

This next set of questions is about sexual experiences you may have had. While some people have had a lot of sexual experience, others have not, so questions may or may not apply to you.

When counting the number of people you have had sex with, please include only people you have had oral, vaginal, or anal sex with. Do not include people that you have only kissed. For these questions, “sex” means that any of these activities happened:

**Vaginal sex** is contact between penis and vagina

**Oral sex** is a mouth or tongue on someone’s vagina, penis, or anus.

**Anal sex** is contact between penis and anus.

Any of these activities will be considered sex for these questions whether condoms were or were not used and whether ejaculation happened or did not happen.

D2. How many people have you had sex with in your entire life? (If 0, skip to section F)

__ __

97 Don't Know

98 Refuse to Answer

99 Not Applicable

D3. How many people have you had sex with in the last 12 months? (If 0, skip to section E)

__ __

97 Don't Know

98 Refuse to Answer

99 Not Applicable

D4. (Ask of Men Only) How many different women have you had sex with since you first began having sex?

__ __ __ __

9997 Don't Know

9998 Refuse to Answer

9999 Not Applicable

***If D4 is equal to 0, then skip to D6.***

D5. (Ask of Men Only) How old were you when you had sex with a woman for the first time?

__ __ __ __

9997 Don't Know

9998 Refuse to Answer

9999 Not Applicable

D6. (Ask of Men Only) How many different men have you had sex with since you first began having sex?

__ __ __ __

9997 Don't Know

9998 Refuse to Answer

9999 Not Applicable

***If D6 is equal to 0, then skip to D8.***

D7. (Ask of Men Only) How old were you when you had sex with a man for the first time?

__ __ __ __

9997 Don't Know

9998 Refuse to Answer

9999 Not Applicable

D8. (Ask of Women Only) How many different men have you had sex with since you first began having sex?

__ __ __ __

9997 Don't Know

9998 Refuse to Answer

9999 Not Applicable

***If D8 is equal to 0, then skip to D10.***

D9. (Ask of Women Only) How old were you when you had sex with a man for the first time?

__ __ __ __

9997 Don't Know

9998 Refuse to Answer

9999 Not Applicable

D10. (Ask of Women Only) How many different women have you had sex with since you first began having sex?

__ __ __ __

9997 Don't Know

9998 Refuse to Answer

9999 Not Applicable

D11. (Ask of Women Only) How old were you when you had sex with a woman for the first time?

__ __ __ __

9997 Don't Know

9998 Refuse to Answer

9999 Not Applicable

D12. How many times have you had vaginal sex in past 12 months. (Choose one)

__ none

__ 1 to 3 times

__ 4 to 10 times

__ more than 10 times

__ Don't Know

__ Refuse to Answer

***If D12 is equal to 0, then skip to D14.***

D13. When you had vaginal sex, how frequently did you use condoms? (Choose one)

__ never

__ sometimes

__ every time

__ Don't Know

__ Refuse to Answer

D14. How many times have you had anal sex in past 12 months. (Choose one)

__ none

__ 1 to 3 times

__ 4 to 10 times

__ more than 10 times

__ Don't Know

__ Refuse to Answer

***If D14 is equal to 0, then skip to section E.***

D15. When you had anal sex, how frequently did you use condoms? (Choose one)

__ never

__ sometimes

__ every time

__ Don't Know

__ Refuse to Answer

D16. How many times have you had insertive anal sex in past 12 months. (Choose one)

__ none

__ 1 to 3 times

__ 4 to 10 times

__ more than 10 times

__ Don't Know

__ Refuse to Answer

***If D16 is equal to 0, then skip to D18.***

D17. When you had insertive anal sex, how frequently did you use condoms? (Choose one)

__ never

__ some times

__ every time

__ Don't Know

__ Refuse to Answer

D18. How many times have you had receptive anal sex in past 12 months. (Choose one)

__ none

__ 1 to 3 times

__ 4 to 10 times

__ more than 10 times

__ Don't Know

__ Refuse to Answer

***If D18 is equal to 0, then skip to instruction before E1.***

D19. When you had receptive anal sex, how frequently did you use condoms? (Choose one)

__ never

__ some times

__ every times

__ Don't Know

__ Refuse to Answer

Section E - Sexual partners risks

This section will ask you questions about the people you have had sex with. Specifically, we will ask you about some of their risks for HIV. Please answer these questions honestly and to the best of your knowledge. Please remember that “sex” means any of these activities happened: **Vaginal sex** (contact between penis and vagina), **Oral sex** (mouth or tongue on someone’s vagina, penis, or anus), **Anal sex** (contact between penis and anus).

E1. To the best of your knowledge, have you ever had sex with anyone who used injected drugs? (This means the drugs were shot into a vein using a needle)? (Choose one)

0 No

1 Yes

7 Don't Know

8 Refuse to Answer

E2. To the best of your knowledge, have you ever had sex with anyone who tested positive for HIV? (Choose one)

0 No

1 Yes

7 Don't Know

8 Refuse to Answer

E3. To the best of your knowledge, have you ever had sex with a man who has had sex with another man? (Choose one)

0 No

1 Yes

7 Don't Know

8 Refuse to Answer

E4. To the best of your knowledge, have you ever had sex with anyone who received a blood transfusion? (Choose one)

0 No

1 Yes

7 Don't Know

8 Refuse to Answer

E5. To the best of your knowledge, have you ever had sex with anyone who has hemophilia? (Choose one)

0 No

1 Yes

7 Don't Know

8 Refuse to Answer

Section F - Alcohol and drug use

Now we are going to ask you some general questions regarding alcohol and drug use. Please remember to answer as honestly as you can.

F1. How often do you drink beer, wine, liquor, or mixed drinks? (Choose one)

0 Never ***Skip to F3***

1 1-3 times per month or less

2 1-2 times per week

3 3-6 times per week

4 Everyday

7 Don't Know

8 Refuse to Answer

F2. On average how many drinks do you have each time you drink?.(One drink is a glass of wine or beer or a mixed drink)

__ __ __ Number of drinks

997 Don't Know

998 Refuse to Answer

F3. Have you ever used any non-injected illegal drugs? These are drugs that are smoked, snorted or taken by mouth. Some examples of non-injected illegal drugs are marijuana (also called, pot), hashish, cocaine (also called, blow or crack), methamphetamines (also called crystal), ecstasy (also called, "e"), mushrooms, and LSD? (Choose one)

0 No ***If no,*** ***skip to F8***

1 Yes

7 Don't Know

8 Refuse to Answer

F4. Please mark all non-injected drugs that you have ever used. (choose one or more of the options)

1. Marijuana (pot)

2. Hashish

3. Cocaine (also called blow or crack)

4. Methamphetamines (crystal)

5. Ecstasy (“e”)

6. Mushrooms

7. LSD

8. Other

***If F4 is not equal to 8, then skip to F5.***

F4a. Please specify the other non-injected drug that you have used.

__ __ __ __ __ __ __ __ __ __ __ __ __ __ __ __ __ __ __ __ __ __ __ __ __ __ __ __ __

F5. When was the first year you used non-injected illegal drugs?

__ __ __ __ yyyy

2097 Don't Know (Year)

2098 Refuse to Answer (Year)

***If F5 is less than B2 then the year that you entered for first year of non-injected drug use is smaller than the year you were born. Please correct the year of your first non-injected drug use and skip to F6.***

F6. When was the last year you used non-injected illegal drugs?

__ __ __ __ yyyy

2097 Don't Know (Year)

2098 Refuse to Answer (Year)

***If F6 is less than B2 then the year that you entered for first year of non-injected drug use is smaller than the year you were born. Please correct the year of your last non-injected drug use and skip to F7***

F7. If you have smoked or snorted illegal drugs, did you share pipes or straws with another person? (Choose one)

1 Always

2 Sometimes

3 Never

7 Don't Know

8 Refuse to Answer

9 Not Applicable

F8. Have you ever used or shot up injection drugs. This means using a needle to inject drugs into a vein. Examples include heroin, cocaine, and amphetamines?

0 No ***Skip to F12***

1 Yes

7 Don't Know

8 Refuse to Answer

F9 Please mark all of the injected drugs you have used. (chose one or more of the options)

- Heroin
- Cocaine
- Amphetamines
- Other

***If F9 is not equal to 4, then skip to F10.***

F9a. Please specify the other injected drug you have used.

__ __ __ __ __ __ __ __ __ __ __ __ __ __ __ __ __ __ __ __ __ __ __ __ __ __ __ __ __

F10. When was the first year you used injected drugs?

__ __ __ __ yyyy

2097 Don't Know (Year)

2098 Refuse to Answer (Year)

***If F10 is less than B2 then THE YEAR THAT YOU ENTERED FIRST YEAR OF INJECTED DRUG USE IS SMALLER THAN THE YEAR YOU WERE BORN. PLEASE CORRECT THE YEAR OF YOUR FIRST INJECTED DRUG USE and skip to F11.***

F11. When was the last year you used injected drugs?

__ __ __ __ yyyy

2097 Don't Know (Year)

2098 Refuse to Answer (Year)

***If F11 is less than F10 then THE YEAR THAT YOU ENTERED FOR THE LAST YEAR OF INJECTED DRUG USE IS SMALLER THAN THE YEAR FOR FIRST USE. PLEASE CORRECT THE YEAR OF YOUR LAST INJECTED DRUG USE and skip to F12.***

F12. Have you ever injected any non-prescription substances including vitamins, anabolic steroids, or hormones? (Choose one)

0 No ***If no, skip to G1***

1 Yes

7 Don't Know

8 Refuse to Answer

F13. Have you ever shared needles or syringes with another person to inject any non-prescriptions substance including drugs, vitamins, anabolic steroids or hormones? (Choose one)

0 No

1 Yes

7 Don't Know

8 Refuse to Answer

Section G- Medical History

In the next set of questions we will ask about some medical treatments you may have had.

G1. Have you ever had a blood transfusion? (Choose one)

0 No ***Skip to G5***

1 Yes

7 Don't Know

8 Refuse to Answer

G2. How many transfusion episodes have you had? __ __

97 Don't Know

98 Refuse to Answer

G3. When was the first year you received a transfusion?

__ __ __ __

9997 Don't Know

9998 Refuse to Answer

***If G3 is less than B2 then THE YEAR THAT YOU ENTERED FOR FIRST YEAR 0F BLOOD TRANSFUSION IS SMALLER THAN THE YEAR THAT YOU WERE BORN. PLEASE CORRECT THE YEAR OF FIRST BLOOD TRANSFUSION and skip to G4.***

G4. What is the name of the place (hospital, Hemocenter or clinic) where this first transfusion happened?

________________________

G5. When was the last year you received a transfusion?

__ __ __ __

9997 Don't Know

9998 Refuse to Answer

***If G5 is less than G3 then THE YEAR THAT YOU ENTERED FOR LAST BLOOD TRANSFUSION IS SMALLER THAN THE YEAR YOU ENTERED FOR FIRST REPORTED BLOOD TRANSFUSION. PLEASE CORREC THE YEAR OF LAST BLOOD TRANSFUSION and skip to G6.***

G6. Have you ever had an organ or tissue transplant?

0 No ***Skip to G8***

1 Yes

7 Don't Know

8 Refuse to Answer

G7. Please mark the type of tissue or organ you had transplanted (choose one or more of the following options)

- Skin (your own skin)
- Skin (another person’s skin)
- Liver
- Heart
- Cornea
- Bone
- Lung
- Kidney
- Stem cell / bone marrow
- Other

***If G7 is not equal to 10, then skip to G8.***

G7a. Please specify the other tissue/organ .

__ __ __ __ __ __ __ __ __ __ __ __ __ __ __ __ __ __ __ __ __ __ __ __ __ __

G8. Have you ever had a surgery? (Choose one)

0 No

1 Yes

7 Don't Know

8 Refuse to Answer

***If G8 is not equal to 1, then skip to G9.***

G8a. Please specify the surgery.

__ __ __ __ __ __ __ __ __ __ __ __ __ __ __ __ __ __ __ __ __ __ __ __ __ __

G9. Have you ever had a tooth extraction? (Choose one)

0 No

1 Yes

7 Don't Know

8 Refuse to Answer

G10. Have you ever had a dental root treated or surgery in your mouth? (Choose one)

0 No

1 Yes

7 Don't Know

8 Refuse to Answer

Section H- Other Potential Risk Factors

H1. Were you born to an HIV positive mother?

0 No

1 Yes

7 Don't Know

8 Refuse to Answer

H2. Were you were breastfed by an HIV positive person?

0 No

1 Yes

7 Don't Know

8 Refuse to Answer

H3. Have you spent three or more nights in jail, prison, or a detention center? (Choose one)

0 No

1 Yes

7 Don't Know

8 Refuse to Answer

H4. Have you had acupuncture treatments? (Choose one)

0 No ***Skip to H6***

1 Yes

7 Don't Know

8 Refuse to Answer

H5. How many times have you had acupuncture treatments? (Choose one)

1 1 time

2 2 to 5 times

3 5 or more times

7 Don't Know

8 Refuse to Answer

H6. How many tattoos do you have on your body? (Choose one)

0 0 (No tattoos) ***Skip to H8***

1 1

2 2

3 3 or more

7 Don't Know

8 Refuse to Answer

H7. Where did you get your most recent tattoo? (Choose one)

1 Tattoo parlor

2 At home, a friends place, or at parties/raves

3 Jail

4 Other

7 Don't Know

8 Refuse to Answer

H8. How many ear or body piercings do you have? (Choose one)

0 0 (No piercings) ***Skip to H10***

1 1

2 2

3 3 or more

7 Don't Know

8 Refuse to Answer

H9. Where did you get your most recent piercing? (Choose one)

1 Pharmacy or medical clinic

2 Tatoo/piercing parlor

3 At home, a friends place, or at parties/raves

4 Jail

5 Other

7 Don't Know

8 Refuse to Answer

Section I

Now, we would like to know about any personal contact you have had with persons who either have AIDS or have tested positive for HIV. In each question, please include only family members, personal friends or acquaintances. (If you are a health care worker, please do NOT include any individuals you have given professional care to, we will ask about those contacts in a minute).

I1. How many people do you personally know who currently have HIV? (Choose one)

0 0 (none) Skip to I3

1 1

2 2 to 4

3 5 or more

7 Don't Know

8 Refuse to Answer

I2. How many people do you personally know with sickle cell and HIV?

0 0 (none)

1 1

2 2 to 4

3 5 or more

7 Don't Know

8 Refuse to Answer

I3. Are you currently working?

0 No ***Skip to J1 (cases) or final screen (control)***

1 Yes

I4. In your profession, do you take care of humans or have exposure to their bodily fluids? (Choose one)

0 No

1 Yes

7 Don't Know

8 Refuse to Answer

I5. In your professional work have you ever had a needle stick injury (accidentally been stuck by a needle or other sharp instrument used for providing medical care to someone else)? (Choose one)

0 No

1 Yes

7 Don't Know

8 Refuse to Answer

I6. In your professional work have you ever had someone else's blood, body fluids, or excrement splashed into your eyes, mouth or in an open skin lesion? (Choose one)

0 No

1 Yes

7 Don't Know

8 Refuse to Answer

9 Not Applicable

Section J - Exposure and Treatment

This is the final section of the questionnaire. It may be difficult for you to respond to these questions, but please remember that we will not tell anyone the answers to the questions.

J1. What is the name of HIV clinic where you are treated for you HIV?

______________________________________________________________________

J2. How do you think you may have become infected with HIV?

__ __ __ __ __ __ __ __ __ __ __ __ __ __ __ __ __ __ __ __ __ __ __ __ __ __ __ __ __ __ __ __ __ __ __ __ __ __ __ __ __ __ __ __ __ __ __ __ __ __

J3. When do you think you may have been infected with HIV? (Year)

__ __ __ __ yyyy

2097 Don't Know (Year)

2098 Refuse to Answer (Year)

J4. When do you think you may have been infected with HIV? (Month) (Choose one)

__ __ January

__ __ February

__ __ March

__ __ April

__ __ May

__ __ June

__ __ July

__ __ August

__ __ September

__ __ October

__ __ November

__ __ December

__ __ Don't Know

__ __ Refuse to Answer

J5. Are you currently taking antiretroviral therapy? (Choose one) 0 No

1 Yes

7 Don't Know

8 Refuse to Answer

J6. Have you taken antiretroviral therapy in the past? (Choose one) 0 No

1 Yes

7 Don't Know

8 Refuse to Answer

Thank you for taking the time to complete this questionnaire. If you have any questions or concerns, please talk to the research assistant or nurse. You can also contact the medical director at our Hemocenter or health clinic

You have finished the questionnaire. From now on, **DO NOT** touch the screen. Please, talk to the research assistant, the person who assisted you at the beginning of this questionnaire. This assistant will close the screen and thank you for your participating in this study.
